# Post-mortem brain histological examination in the substantia nigra and subthalamic nucleus in Parkinson’s disease following deep brain stimulation

**DOI:** 10.3389/fnins.2022.948523

**Published:** 2022-09-14

**Authors:** Srestha Mazumder, Anita Y. Bahar, Claire E. Shepherd, Asheeta A. Prasad

**Affiliations:** ^1^School of Psychology, University of New South Wales, Sydney, NSW, Australia; ^2^Neuroscience Research Australia, Sydney, NSW, Australia; ^3^School of Medicine, University of New South Wales, Sydney, NSW, Australia; ^4^Faculty of Medicine and Health, School of Medical Sciences, University of Sydney, Sydney, NSW, Australia

**Keywords:** Parkinson’s disease, deep brain stimulation, subthalamic nucleus, substantia nigra, alpha-synuclein

## Abstract

Parkinson’s disease (PD) is a progressive neurodegenerative disorder, pathologically hallmarked by the loss of dopamine neurons in the substantia nigra (SN) and alpha-synuclein aggregation. Deep brain stimulation (DBS) of the subthalamic nucleus (STN) is a common target to treat the motor symptoms in PD. However, we have less understanding of the cellular changes in the STN during PD, and the impact of DBS on the STN and SN is limited. We examined cellular changes in the SN and STN in PD patients with and without STN-DBS treatment. Post-mortem brain tissues from 6 PD non-STN-DBS patients, 5 PD STN-DBS patients, and 6 age-matched controls were stained with markers for neurodegeneration (tyrosine hydroxylase, alpha-synuclein, and neuronal loss) and astrogliosis (glial fibrillary acidic protein). Changes were assessed using quantitative and semi-quantitative microscopy techniques. As expected, significant neuronal cell loss, alpha-synuclein pathology, and variable astrogliosis were observed in the SN in PD. No neuronal cell loss or astrogliosis was observed in the STN, although alpha-synuclein deposition was present in the STN in all PD cases. DBS did not alter neuronal loss, astrogliosis, or alpha-synuclein pathology in either the SN or STN. This study reports selective pathology in the STN with deposits of alpha-synuclein in the absence of significant neuronal cell loss or inflammation in PD. Despite being effective for the treatment of PD, this small post-mortem study suggests that DBS of the STN does not appear to modulate histological changes in astrogliosis or neuronal survival, suggesting that the therapeutic effects of DBS mechanism may transiently affect STN neural activity.

## Introduction

First described over 200 years ago, Parkinson’s disease (PD) is a neurodegenerative disorder characterized by debilitating tremor, rigidity, and bradykinesia (DeMaagd and Philip, 2015). PD is pathologically characterized at post-mortem by the abnormal deposition of alpha-synuclein in the form of Lewy bodies and Lewy neurites (Braak et al., 1999) that deposit in a progressive pattern throughout the brainstem, limbic, and neocortical brain regions (Braak et al., 2003). Increased neuroinflammation and neuronal cell loss are also prominent, particularly in the substantia nigra (SN) (Fearnley and Lees, 1991; Hardman et al., 1996; Hirsch et al., 2003). Currently, there are no treatments to halt or slow disease progression but deep brain stimulation (DBS) of the subthalamic nucleus (STN-DBS) is highly effective at relieving motor symptoms in PD (Limousin et al., 1995; Tagliati et al., 2010; Limousin and Foltynie, 2019; Jakobs et al., 2019). The irregular burst of neuronal firing in the STN in PD patients is destabilized by DBS (Bergman et al., 1994; Remple et al., 2011) but less is known about the STN pathology in PD or how this is impacted by DBS treatment.

Limited preclinical studies in rodents and non-human primates suggest that DBS may alter disease progression through a combination of neuroinflammatory and neurodegenerative changes (Temel et al., 2006; Wallace et al., 2007). Specifically, animal models show that STN-DBS decreases neuroinflammation expressed through microglia and astrocytes in the SN and slows down the loss of dopamine neurons (Charles et al., 2008; Spieles-Engemann et al., 2010; Tawfik et al., 2010; Vedam-Mai et al., 2012). However, there are only two post-mortem comparative neuropathology studies of PD patients with and without STN-DBS. Pal et al. (2017) carried out a semi-quantitative assessment of SN depigmentation and alpha-synuclein pathology and reported that STN-DBS subjects have higher alpha-synuclein density scores, but do not display differences in SN depigmentation. The study did not examine the STN and did not investigate changes in inflammation. In contrast, Pienaar et al. (2015) investigated microvascular integrity in PD patients with and without STN-DBS and reported significant vascular changes and lowered microglial activation following STN-DBS. Our study provides a quantitative analysis of cell loss, alpha-synuclein pathology, and inflammation in the STN and SN in PD cases with and without STN-DBS compared with controls.

## Materials and methods

### Cases

Formalin-fixed, paraffin-embedded 10 μm serial sections were obtained from the Sydney Brain Bank (SBB). Tissue was taken from the SN at the level of the red nucleus and at the most posterior level of the STN from 6 PD patients without DBS, 5 PD patients with STN-DBS, and 6 age-matched controls. The STN was delineated on an H&E-stained section as previously described in Pienaar et al. (2015). Briefly, the STN was identified as a compact nucleus located medial to the internal capsule and superolateral to the SN on coronal slices.

The research was carried out under UNSW Human Research Ethics Committee (HREC) approval (project no. HC180835). All PD cases met neuropathological criteria for PD with no coexisting disease (Dickson et al., 2009). PD STN-DBS cases were chosen on the basis of having an implanted electrode in the STN during life. All control subjects were free from neuropathological and clinical neurodegenerative or neuropsychiatric diseases.

### Immunohistochemistry

Immunohistochemistry to detect astrocytes [anti-glial fibrillary acidic protein (GFAP)] and dopamine neurons (anti-TH) was carried out using the Novolink polymer detection system (Leica Biosystems #RE7150-K). Briefly, serial sections were deparaffinized in xylene and then rehydrated in serial ethanol dilutions. Sodium citrate buffer (0.1 M, pH 6) was used for antigen retrieval. Antigen retrieval was performed by placing the sections in boiling sodium citrate buffer for 3 min. Sections were then left to cool for 30 min before washing the slides in deionized water. Endogenous peroxide was neutralized using Novolink peroxide block for 5 min before the slides were washed in 0.1 M Tris-buffered saline (TBS, pH 7.3). Sections were then incubated for a further 5 min with Novolink protein block followed by three TBS washes. Primary antibodies anti-tyrosine hydroxylase (AB112, Abcam, 1:750) and anti-GFAP (AB7260, Abcam, 1:2,000) were diluted in TBS and added individually to sections placed in a humid box and incubated at 4°C overnight. On day 2, sections were washed in TBS before being incubated for 30 min in Novolink Polymer (Anti-rabbit Poly-HRP-IgG (<25 μg/ml) containing 10% (v/v) animal serum in TBS/0.09% ProClinTM 950) followed by TBS washes. Peroxide activity was developed using 3,3′-diaminobenzidine (DAB) working solution on all sections for 5 min followed by a rinse with tap water. Slides were then counterstained with hematoxylin for 3 min before a 5 min wash under running tap water. Prior to cover slipping, all sections were dehydrated in ethanol (70%, 95%, and 100%) before being placed in xylene. Sections were cover slipped with Entellan (ProSciTech, Kirwin, QLD, Australia).

Immunohistochemistry for alpha-synuclein was performed on a Discovery XT (Ventana Medical Systems Inc, Tucson, Arizona) autostainer using OmniMap and ChromoMap multimer technology detection systems. Antigen retrieval was performed for 30 min using cell conditioning 1 pretreatment solution before adding purified mouse anti-alpha-synuclein (610787, BD Biosciences-US, 1:7000). Slides were incubated for 1 h prior to counterstaining with hematoxylin and cover slipping with Entellan.

Neurons in the STN and SN were identified with H&E stain. Sections were deparaffinized in xylene two times for 3 min followed by rehydration in 100% alcohol two times for 3 min. Sections were then transferred to 95% alcohol for 3 min followed by 70% alcohol for another 3 min before being placed into distilled water for 3 min. Sections were incubated in hematoxylin for 3 min followed by a rinse under running tap water. Sections were then incubated with lithium carbonate for 3 min and then washed under tap water. Eosin was added to the tissue section for 3 min before the slides were placed in 100% alcohol wash for 3 min two times followed by xylene for 3 min two times. Slides were then cover slipped with Entellan.

### Quantitation

Slides were visualized on the Olympus BX51 microscope and captured using Zeiss AxioLab light microscope and software at 200× magnification. All assessments were performed by an investigator blinded to case details. Ten representative images were taken from one section per case, and from these, three images were chosen at random for quantitation. Immunoreactive cells were quantified using ImageJ.

In both the SN and STN, neurons were clearly identified by the presence of a clear cytoplasm and nucleolus. Tyrosine hydroxylase (TH) immunoreactivity was observed in the cell soma and proximal axon of neurons in the SN (Kordower et al., 2013). The number of neurons in each 200× field was counted, and the average was calculated.

Astrocytes were clearly identified by GFAP immunoreactivity in the cellular processes surrounding a nucleus. The total number of astrocytes in each 200× field was counted using ImageJ, and the average of the 3 fields was calculated.

Alpha-synuclein deposits were identified in the form of alpha-synuclein-positive Lewy bodies, Lewy neurites, and glial inclusion (Pal et al., 2017). ImageJ software was used to identify alpha-synuclein immunolabeling, and the average areal fraction of the 3 fields was calculated.

### Statistics

IBM SPSS Statistics software 27 was used to carry out all statistical analyses. A power analysis was carried out using PASS software to calculate the sample size required to detect various effect sizes with 80% power. With groups of 5 cases, there was at least an 80% chance of detecting large differences (where mean differences were between 18 and 24 and standard deviations of less than 11). Non-parametric Kruskal–Wallis test statistics was used to assess differences between the three groups. Significance values were adjusted by the Bonferroni correction for multiple tests.

## Results

### Cases

Details of all cases, including age, gender, and disease duration, are provided in Table 1. All cases were matched for age (*p* = 0.17) and post-mortem delay (*p* = 0.69). Braak Lewy body stage (*p* = 0.89) and disease duration (*p* = 0.22) were not significantly different between PD and STN-DBS cases. These variables were therefore not considered further.

**TABLE 1:** Demographic and clinical characteristics of all cases included.

Parameters	Control (*n* = 6)	PD (*n* = 6)	PD STN-DBS (*n* = 5)
Sex ratio (M:F)	6:0	6:0	3:2
Age at death (years)	81.33 ± 3.13	76.833 ± 1.86	74.40 ± 2.20
Disease duration (years)	N/A	14.667 ± 2.56	21.400 ± 2.83
Post-mortem delay (hours)	20.833 ± 3.84	16.167 ± 2.71	21.200 ± 5.68
Duration of STN-DBS (years)	N/A	N/A	10.2 ± 1.69
Braak Lewy body stage	N/A	V (4) VI (2)	V (3) VI (2)

Results are shown as the mean ± SEM. N/A, not applicable.

#### Analysis of cell loss and astrogliosis in the substantia nigra

**FIGURE 1:**
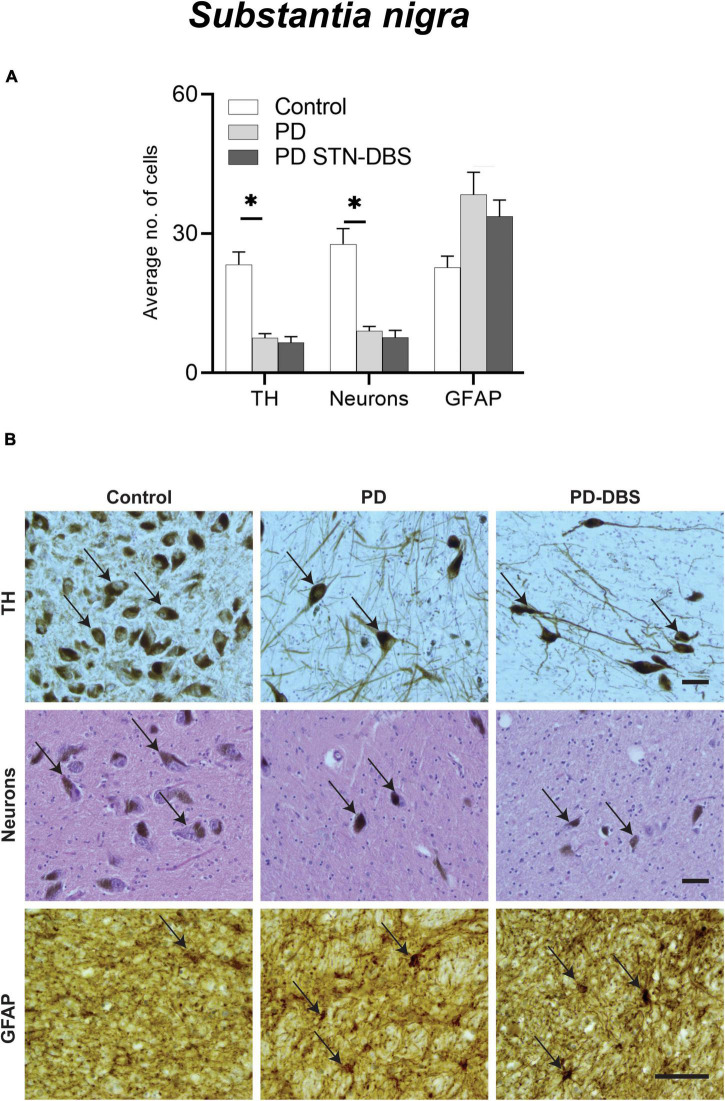
Quantitation of neuronal loss and astrogliosis in the substantia nigra (SN) of control, Parkinson’s disease (PD) and PD STN-DBS cases. **(A)** Significant differences between control and PD cases were observed in neuronal loss (*p* < 0.018), TH neurons (*p* < 0.015), and astrogliosis (GFAP: *p* = 0.028). Comparison of PD patients with STN-DBS and PD patients without STN-DBS showed no differences in neuronal loss (*p* = 1.000), TH neurons (*p* = 1.000), or inflammatory marker (GFAP: *p* = 1.000). **(B)** Representative photomicrographs of TH (black arrows showing dopamine neurons), neuronal density identified by hematoxylin and eosin (black arrows showing H&E stained neurons), GFAP (black arrow showing astrocyte) in control, PD, and PD STN-DBS in the SN. All images were taken at 200× magnification. All values are expressed as mean ± SEM. * Indicates *p* < 0.050. Scale bar = 50 microns.

Means and SEM of cellular counts are presented in Table 2. Compared with controls, both PD groups showed a significant decrease in total (PD no DBS *p* = 0.018, PD STN-DBS *p* = 0.009) and TH-positive neurons (PD no DBS *p* = 0.015, PD STN-DBS *p* = 0.011) in the SN with *no difference* between PD groups (total neurons *p* = 1.000, TH-positive neurons *p* = 1.000). There was a significant increase in the number of astrocytes in the PD group compared with controls (*p* = 0.028) but no significant increase in GFAP-positive astrocytes in STN-DBS compared with controls (*p* = 0.127) and no difference between PD and DBS group (*p* = 1.000) (Figures 1A,B).

**TABLE 2:** Means and SEM of cellular analysis in substantia nigra (SN) and subthalamic nucleus (STN).

Brain region	Positive cell type	Control (*n* = 6)	PD (*n* = 6)	PD STN-DBS (*n* = 5)
Substantia nigra	Dopamine neurons (TH)	23.33 ± 2.69	7.50 ± 0.95*	6.60 ± 1.20*
	Cells (H&E)	27.72 ± 3.43	9.06 ± 0.96*	7.67 ± 1.48*
	Astrocytes (GFAP)	22.67 ± 2.46	38.39 ± 4.8*	33.66 ± 3.54*
	Alpha-synuclein	0.00 ± 0.00	1.50 ± 0.22*	1.60 ± 0.24*
Subthalamic nucleus	Cells (H&E)	17.06 ± 3.96	11.50 ± 1.02	11.87 ± 2.45
	Astrocytes (GFAP)	32.86 ± 4.37	30.70 ± 1.10	29.60 ± 5.15
	Alpha-synuclein	0.00 ± 0.00	1.33 ± 0.21*	1.60 ± 0.24*

*p < 0.05 versus control.

#### Analysis of cell loss and inflammation in the subthalamic nucleus

**FIGURE 2:**
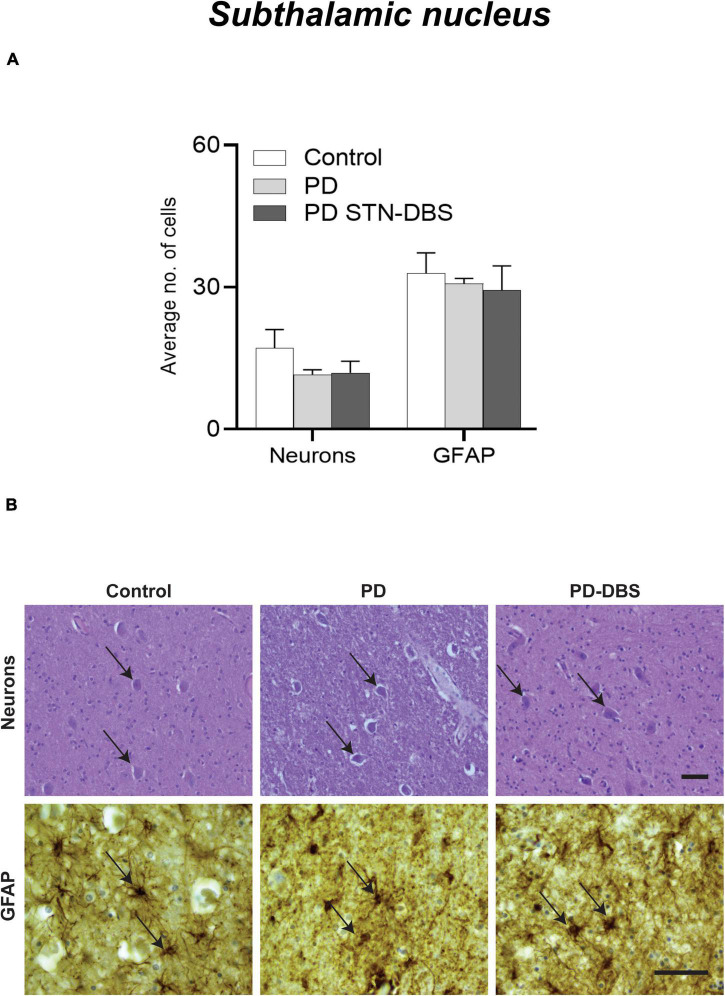
Quantitation of neuronal loss and astrogliosis in the subthalamic nucleus (STN) of control, Parkinson’s disease (PD) and PD STN-DBS cases. **(A)** Between control and PD patients no significant difference was observed in neuronal (*p* = 0.358) or inflammatory marker (GFAP: *p* = 0.612). **(B)** Representative photomicrographs of neuronal density (black arrow showing H&E stained neuron), GFAP (black arrow showing astrocyte) in control, PD, and PD STN-DBS patients in the STN. All values are expressed as mean ± SEM. Scale bar = 50 microns.

In contrast to the SN, there was no significant difference in neuronal density (*p* = 0.358) or astrocytes (*p* = 0.612) in the STN between any groups (Table 2 and Figures 2A,B).

#### Alpha-synuclein pathology

**FIGURE 3:**
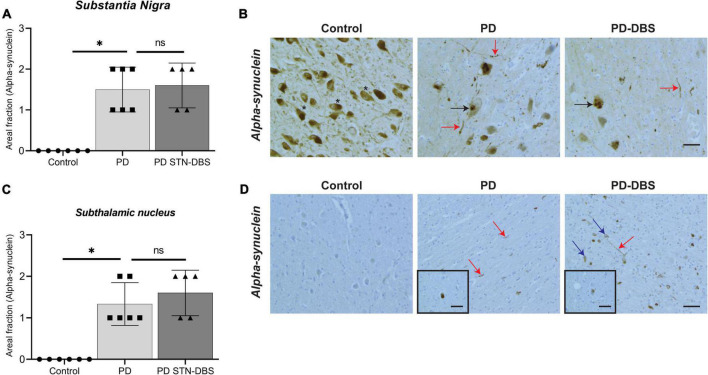
Quantitation of alpha-synuclein pathology in the substantia nigra (SN) and subthalamic nucleus (STN) of control, Parkinson’s disease (PD) and PD STN-DBS cases. **(A)** No alpha-synuclein pathology was seen in the SN control cases. Alpha-synuclein pathology was significantly higher in PD (*p* = 0.008) and STN-DBS cases (*p* = 0.007). No changes in alpha-synuclein expression were seen in the SN in PD and STN-DBS patients (*p* = 1.000). **(B)** Asterisks indicate pigmented neurons in the SN. Both Lewy bodies (black arrows) and Lewy neurites (red arrows) were observed in the SN PD and Deep brain stimulation (DBS) cases. **(C)** In the control cases, no alpha-synuclein was present in the STN. Alpha-synuclein aggregates were significantly higher in PD (*p* = 0.013) and STN-DBS (*p* = 0.004) compared to controls with no differences between PD and STN-DBS cases (*p* = 1.000). **(D)** Lewy neurites (red arrows) and Lewy bodies (blue arrows) were observed in the STN in all PD cases (inserts in D). Occasional Lewy bodies and alpha-synuclein positive neurons were also seen (D inserts). All values are expressed as mean ± SEM. Scale bar = 50 microns.

Consistent with diagnosis, significantly greater alpha-synuclein pathology was seen in the SN of PD cases compared with controls (PD no DBS *p* = 0.008, PD STN-DBS *p* = 0.007, Table 2) with no differences between PD groups (*p* = 1.000, Table 2 and Figures 3A,B). The majority of alpha-synuclein pathology was in the form of Lewy neurites with occasional Lewy bodies present in the SN (see Figure 3B). Alpha-synuclein was also significantly increased in the STN in PD cases compared with controls (PD no DBS *p* = 0.013, PD STN-DBS *p* = 0.004) with no difference between PD and DBS groups (*p* = 1.000). Alpha-synuclein was predominantly observed in the form of neurites and glial inclusions in the STN (Figure 3D), although occasional Lewy bodies and alpha-synuclein-positive neurons were also seen (Figure 3D inserts).

## Discussion

This is the first study to carry out a quantitative assessment of cell loss, alpha-synuclein pathology, and inflammation in the SN and the STN of PD patients with and without STN-DBS. Consistent with diagnosis, our study confirms significant loss of total and TH-positive neurons (69% of dopamine and 70% of total cells lost) and an increase in alpha-synuclein in the SN in PD. In contrast, despite the presence of significant alpha-synuclein pathology, we did not observe any loss of neurons or astrogliosis in the STN of our PD cases. Importantly, no loss of total or dopaminergic neurons, or changes in astrogliosis or alpha-synuclein deposition were observed following DBS, suggesting that STN-DBS may not exert its therapeutic effect *via* changes in neuronal loss, astrogliosis, or alpha-synuclein.

Our findings do not support previous animal studies showing that DBS may alter disease progression through decreased neuroinflammation and reduced dopamine cell loss (Wallace et al., 2007; Charles et al., 2008; Spieles-Engemann et al., 2010; Tawfik et al., 2010). Rather, they are consistent with human imaging studies showing that striatal dopamine levels are not affected following STN-DBS (Hilker et al., 2003; Strafella et al., 2003; Thobois et al., 2003). Although there are limited human brain tissue studies examining cellular changes following STN-DBS, one study has demonstrated neurogenesis in the subventricular zone, suggesting that DBS may be capable of increasing cellular plasticity even at sites remote from the electrode location (Vedam-Mai et al., 2014). Neuroprotective molecules such as BDNF have also been shown to be upregulated in the rodent basal ganglia post-DBS (Fischer et al., 2017; Fischer and Sortwell, 2018). Although our studies suggest that this is unlikely to prevent neuronal cell death in the human SN, an elegant study using optogenetics to elucidate the target cell types underlying DBS suggests that the therapeutic effects can be accounted for by selective stimulation of afferent axons, indicating that disruption of circuit loops could represent a common pathway for treatment (Gradinaru et al., 2009). Indeed, clinical studies show a significant increase in motor dysfunction with overnight “off” STN-DBS conditions compared with “on” state (Kojovic et al., 2019). A single pulse of STN-DBS has been recorded between 1 and 400 ms after stimulation (Baker et al., 2002), indicating DBS is linked to short-term changes in electrophysiological activity. In PD patients, electrophysiological recording show signature enhanced neural firing in the STN (Hammond et al., 2007) which is reduced by DBS (Eusebio et al., 2011). These studies and the findings from our histological analysis indicate that the therapeutic effects of DBS are transient and most likely occur through “inhibition,” “excitation,” or “disruption” of the cortico-basal ganglia loop (Chiken and Nambu, 2016).

Although gliosis is a normal response to brain injury and is commonly observed in neurodegenerative disorders such as Alzheimer’s disease, reports of astrogliosis in the SN of PD patients are conflicting (Urbanc et al., 2002). Indeed, some post-mortem studies have reported mild increases in GFAP-positive astrocytes in the SN in PD (Mirza et al., 2000; McGeer and McGeer, 2008), while others have reported that the number and morphology of astrocytes remain unchanged (Knott et al., 1999; Tong et al., 2015). While our study did identify an increase in astrogliosis in the SN of our non-DBS PD cases, no significant change was seen in the SN of the STN-DBS cases, and no astrogliosis was present in the STN of either PD group, despite significant alpha-synuclein deposition. This is interesting, as primate studies suggest that DBS reduces glutamate toxicity (Wallace et al., 2007) and/or astrocyte-mediated abolition of spontaneous spindle oscillations (Hardman et al., 1996, 1997; Temel et al., 2006; Wallace et al., 2007) and may lower neuroinflammation. Recent studies also suggest that there is a relationship between astrocytic dysfunction and alpha-synuclein accumulation in PD (Tong et al., 2015) but this was certainly not observed in the STN in our study. It is clear that the role of astrocytes in PD is likely highly complex, and changes in astrocyte function are not easily captured using generic markers such as GFAP. Indeed, A1 astrocytes have been shown to be neurotoxic in PD (Hendrickx et al., 2017) and are involved in an intimate relationship with microglia to control the inflammatory response (Röhl and Sievers, 2005; Jo et al., 2017; Liddelow et al., 2017). To determine whether STN-DBS exerts a therapeutic effect *via* modulating neuroinflammation, future studies should utilize glial markers that recognize the morphologically and functionally diverse states of both astrocytes and microglia. Analysis of inflammatory cytokines and chemokines would also be informative. Although these studies would require access to frozen brain tissue, which is seldom available from DBS cases due to the need for whole brain fixation to delineate the electrode tract and termination point.

In contrast to Pal et al. (2017), we did not observe an increase in alpha-synuclein pathology in the SN following STN-DBS. Although differences in the methods of analysis and cohort size may explain the discrepancy between these studies, differences in the methods used for alpha-synuclein detection may also be relevant. Indeed, Pal et al. (2017) used Thioflavin S staining to identify Lewy bodies within the SN, whereas we carried out alpha-synuclein immunohistochemistry on a Ventana stainer (see Section “Materials and methods”), which is considered the gold standard for Lewy body and Lewy neurite detection (Beach et al., 2008). Alpha-synuclein pathology in the STN has not been well investigated in PD with or without DBS. However, our findings are consistent with a previous case study that reported Lewy body formation in the STN in a PD case without DBS (Ohama and Ikuta, 1976). Most of the alpha-synuclein pathology we observed was in the form of Lewy neurites, although occasional Lewy bodies and neuronal cytoplasmic staining were seen (see Figure 3), which may also be attributable to the sensitive methods of immunohistochemical detection that were used in this study. Lewy neurites in axons appear prior to Lewy bodies and represent some of the earliest pathology seen in the PD brainstem (Braak et al., 1999, 2003). The predominance of Lewy neurites in PD cases both with and without DBS in the absence of significant neuronal loss suggests that the STN is affected later in the disease process as it may be more resistant to neurodegeneration in PD.

A significant limitation of this study is the number of cases that were available for analysis. However, access to well-characterized brain tissue from PD cases with DBS is limited, and we utilized all of the available cases in the SBB. While we could have increased the number of PD and control cases examined, we do not believe this would significantly impact our results as our observations in the PD without DBS and control cases were representative of findings from previous studies (Pienaar et al., 2015).

In conclusion, our findings demonstrate no significant effect of DBS on total or dopaminergic cell loss, alpha-synuclein pathology, or astrogliosis in the SN and STN. The absence of neuronal loss is consistent with other clinical and imaging studies indicating that STN-DBS may not be neuroprotective and most likely has a transient therapeutic effect (Lilleeng et al., 2014). While we did not observe a change in GFAP-positive astrogliosis in STN-DBS, it is widely acknowledged that PD-related neuroinflammation is a complex process, and we did not undertake a thorough analysis of glial subtypes or inflammatory modulators. Future studies should therefore address this limitation.

## Data availability statement

The raw data supporting the conclusions of this article will be made available by the authors, without undue reservation.

## Ethics statement

This research project was approved by the Human Research Ethics Committees of the University of New South Wales (HREC project no. HC180835). Brain tissues were obtained from the Sydney Brain Bank that holds ethical approval and informed consent from the participants for brain banking through UNSW HREC 200026.

## Author contributions

SM contributed to writing – original draft, review, and editing, project administration, investigation, data curation, visualization, and formal analysis. AB was involved in the data curation, visualization, and formal analysis. CS and AP contributed to the conceptualization, methodology, software, validation, formal analysis, writing – review and editing, supervision, and project administration. All authors contributed to the article and approved the submitted version.
